# Heparan sulfate negatively regulates intestinal stem cell proliferation in *Drosophila* adult midgut

**DOI:** 10.1242/bio.047126

**Published:** 2019-10-15

**Authors:** Hubing Ma, Huiqing Zhao, Fuli Liu, Hang Zhao, Ruiyan Kong, Lin Shi, Min Wei, Zhouhua Li

**Affiliations:** College of Life Sciences, Capital Normal University, Beijing 100048, China

**Keywords:** Intestinal stem cell, Heparan sulfate, *Drosophila*, Tissue homeostasis, Dpp signaling

## Abstract

Tissue homeostasis is maintained by differentiated progeny of residential stem cells. Both extrinsic signals and intrinsic factors play critical roles in the proliferation and differentiation of adult intestinal stem cells (ISCs). However, how extrinsic signals are transduced into ISCs still remains unclear. Here, we find that heparan sulfate (HS), a class of glycosaminoglycan (GAG) chains, negatively regulates progenitor proliferation and differentiation to maintain midgut homeostasis under physiological conditions. Interestingly, HS depletion in progenitors results in inactivation of Decapentaplegic (Dpp) signaling. Dpp signal inactivation in progenitors resembles *HS-deficient* intestines. Ectopic Dpp signaling completely rescued the defects caused by HS depletion. Taken together, these data demonstrate that HS is required for Dpp signaling to maintain midgut homeostasis. Our results provide insight into the regulatory mechanisms of how extrinsic signals are transduced into stem cells to regulate their proliferation and differentiation.

## Abstract (precis)

**Summary:** Heparan sulfate chains in *Drosophila* adult intestinal stem cells negatively regulate stem cell proliferation and differentiation to control tissue homeostasis under physiological conditions.

## INTRODUCTION

Adult stem cells are responsible for tissue homeostasis in the tissues in which they reside; frequently lost cells are constantly replenished by the progeny of stem cells. The proliferation and differentiation of adult stem cells must be tightly balanced. Disruption of this balance will lead to either excessive stem cells or stem cell depletion, eventually resulting in various diseases, such as cancer (Lin, 2008; Morrison and Spradling, 2008; Radtke and Clevers, 2005; Xie and Spradling, 1998). Therefore, understanding of the underlying mechanisms controlling adult stem cell proliferation and differentiation will provide insight into the potential development of therapeutic applications for human diseases.

The posterior midgut of the adult *Drosophila* intestine is an excellent system to study how stem cell proliferation and differentiation are regulated. Mammalian and *Drosophila* intestines show marked similarities in terms of development, cellular make-up and genetic control (Casali and Batlle, 2009; Edgar, 2012; Stainier, 2005; Wang and Hou, 2010). Adult intestinal stem cells (ISCs) are interspersed along the base membrane of the *Drosophila* adult midgut (Micchelli and Perrimon, 2006; Ohlstein and Spradling, 2006). Initial studies proposed that ISCs constantly undergo asymmetric divisions and produce non-dividing enteroblasts (EBs) (Micchelli and Perrimon, 2006; Ohlstein and Spradling, 2006). The ligand of the Notch pathway, Delta (Dl), is specifically expressed in ISCs, while Notch receptor is expressed in both ISCs and EBs. ISCs signal via Dl to activate Notch signaling in EBs (Ohlstein and Spradling, 2007). EBs terminally differentiate into either an absorptive enterocyte (EC) or a secretory enteroendocrine cell (ee) depending on their signaling environments (Beebe et al., 2010; Micchelli and Perrimon, 2006; Ohlstein and Spradling, 2007; Perdigoto et al., 2011; Yeung et al., 2011). Recent studies demonstrate that in response to differentiation and subsequent loss of a neighboring ISC (or vice versa), a significant proportion of ISCs divide symmetrically (de Navascués et al., 2012; Goulas et al., 2012; O'Brien et al., 2011). Moreover, ee cells may not be generated from EBs, but directly from ISCs or ee progenitor cells (EEPs) (Biteau and Jasper, 2014; Chen et al., 2018; Zeng et al., 2015). Interestingly, unlike in other systems in which differentiated cells can de-differentiate into stem cells, we found that no regeneration of new ISCs could be observed after all the progenitors were ablated in the intestines, indicating that fully differentiated cells are likely unable to de-differentiate into ISCs when all the progenitors are depleted (Brawley and Matunis, 2004; Lu and Li, 2015; Raff, 2003).

Numerous studies have shown that ISC proliferation and differentiation under physiological conditions and during tissue regeneration are regulated by many signaling pathways and intrinsic factors, including the Notch, Wingless (Wg), Janus Kinase/Signal Transducer and Activator of Transcription (JAK/STAT), Epidermal Growth Factor Receptor (EGFR), Hippo (Hpo), Insulin, Hedgehog (Hh) and Bone Morphogenetic Protein (BMP) signaling pathways (Amcheslavsky et al., 2009; Biteau and Jasper, 2011; Buchon et al., 2009; Chakrabarti et al., 2016; Chen et al., 2016; Choi et al., 2011; Cordero et al., 2012; Guo and Ohlstein, 2015; Han et al., 2015; Jiang et al., 2011, 2009; Jin et al., 2017; Karpowicz et al., 2010; Lee et al., 2009; Li et al., 2013a,b, 2014; Lin and Xi, 2008; Lin et al., 2008; Martorell et al., 2014; Ohlstein and Spradling, 2006, 2007; Rahman et al., 2017; Ren et al., 2010, 2015; Schell et al., 2017; Shaw et al., 2010; Singh et al., 2016; Staley and Irvine, 2010; Tian and Jiang, 2014; Tian et al., 2015, 2017; Xu et al., 2011; Zhai et al., 2017; Zhou et al., 2015). However, it remains unclear how extrinsic signals are transduced into ISCs to regulate their proliferation and differentiation under physiological conditions.

Heparan sulfate chains are attached to the core protein of heperan sulfate proteoglycans (HSPGs), macromolecules presented on the cell surface and in the extracellular matrix (ECM). There are three evolutionarily conserved families of HSPGs: Glypicans and Syndecans are two major cell surface HSPGs, while Perlecans are secreted HSPGs that are mainly distributed in the ECM (Esko and Lindahl, 2001; Esko and Selleck, 2002; Lin, 2004). HS chain biosynthesis is initiated in the Golgi apparatus at the GAG attachment site(s) of the core protein. HS is synthesized by a series of conserved HS biosynthetic and modifying enzymes, including Gal transferases, the exostosin (EXT) proteins [Tout-velu (Ttv), Sister of ttv (Sotv), Brother of ttv (Botv)], Sulfateless (Sfl) and Sugarless (Sgl) (Esko and Lindahl, 2001; Esko and Selleck, 2002; Lin, 2004). Previous studies demonstrate that HSPGs are required for the distribution of several well-known morphogens, including Wg, Hh, Upd and Dpp (Belenkaya et al., 2004; Bellaiche et al., 1998; Binari et al., 1997; Bornemann et al., 2004; Dani et al., 2012; Filmus et al., 2008; Fujise et al., 2003; Jackson et al., 1997; Kamimura et al., 2006; Levings and Nakato, 2017; Lin and Perrimon, 1999, 2000, 2002; Liu et al., 2010; Mii et al., 2017; Takei et al., 2004; Yan and Lin, 2009; Yu et al., 2017; Zhang et al., 2013). Although HS plays important roles in the functions of HSPGs, its role(s) in regulating ISC proliferation and differentiation under physiological conditions remains elusive.

In this study, we provide evidence that HS in progenitors restricts ISC proliferation and differentiation under normal homeostasis. Importantly, we demonstrate that HS is required for Dpp signal activation. Thus, our data uncover a mechanism of HS to maintain midgut homeostasis.

## RESULTS

### Loss of HS in progenitors leads to disruption of midgut homeostasis

**Fig. 1.**
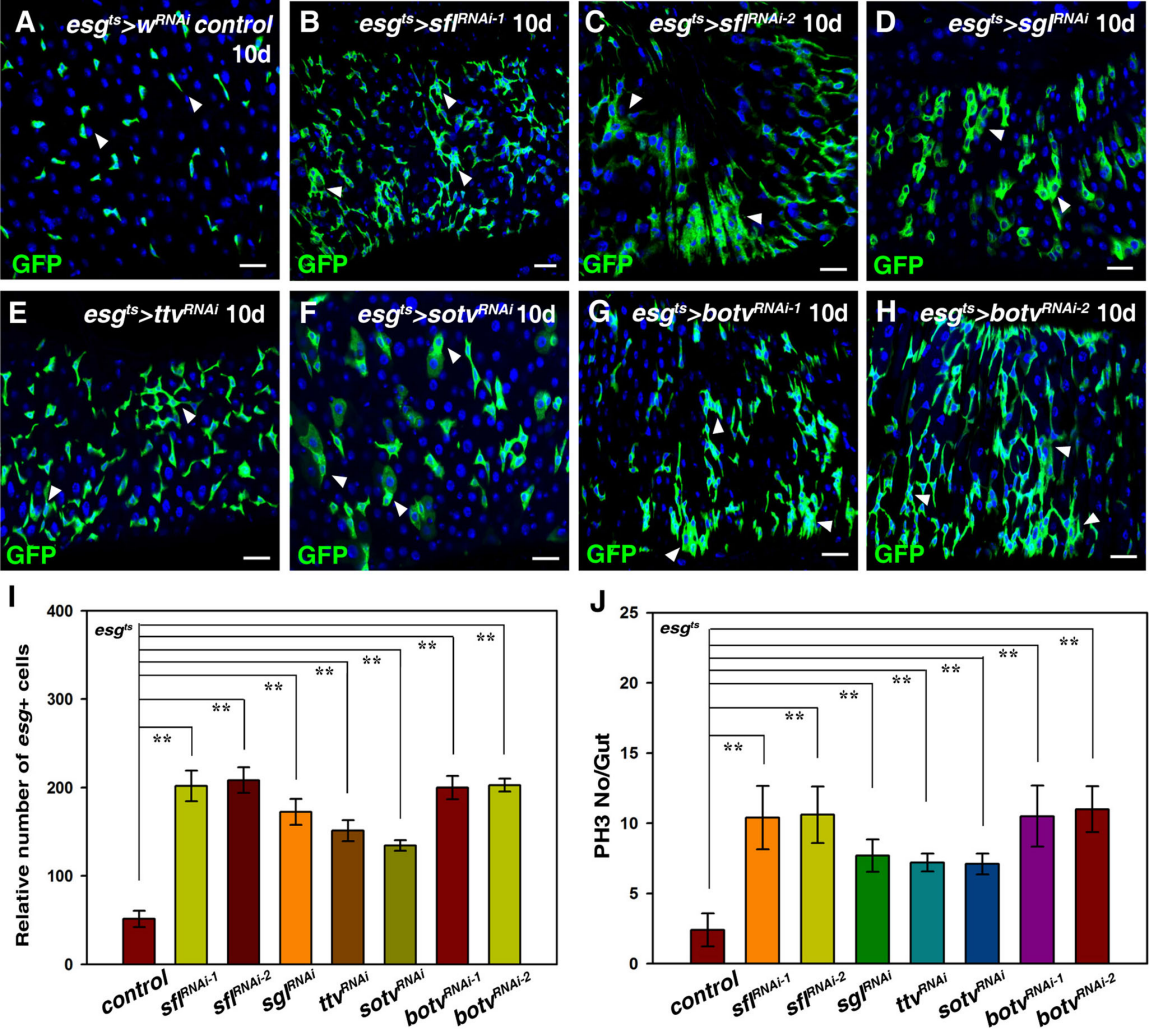
**HS in progenitors restricts ISC proliferation.** (A) *esg*^+^ cells (green) in control flies at 29°C for 10 days (white arrowheads). (B,C) The number of *esg*^+^ cells (green) is dramatically increased in *esg^ts^>sfl^RNAi^* flies at 29°C for 10 days (white arrowheads). (D) The number of *esg*^+^ cells (green) is dramatically increased in *esg^ts^>sgl^RNAi^* flies at 29°C for 10 days (white arrowheads). (E) The number of *esg*^+^ cells (green) is dramatically increased in *esg^ts^>ttv^RNAi^* flies at 29°C for 10 days (white arrowheads). (F) The number of *esg*^+^ cells (green) is dramatically increased in *esg^ts^>sotv^RNAi^* flies at 29°C for 10 days (white arrowheads). (G,H) The number of *esg*^+^ cells (green) is dramatically increased in *esg^ts^>botv^RNAi^* flies at 29°C for 10 days (white arrowheads). (I) Quantification of the relative number of *esg^+^* cells in the different genotypes indicated. mean±s.d. is shown. *n*=10–15 intestines. **P<0.01. (J) Quantification of the number of pH3 per gut in the different genotypes indicated. mean±s.d. is shown. *n*=10–15 intestines. **P<0.01. In all panels except graphs, GFP is in green and blue indicates DAPI staining for DNA. Scale bars: 20 μm.

In order to identify intrinsic factors regulating the proliferation and differentiation of ISCs, we carried out a genome-wide RNAi screen using the *esgGal4, UAS-GFP, tubGal80^ts^* (*esg^ts^*) driver in the posterior midgut. *esgGal4* is expressed in progenitors (ISCs and EBs) in the midgut. 11,316 RNAi lines from Vienna *Drosophila* RNAi Center (VDRC), Fly stocks of National Institute of Genetics (NIG-FLY), and the Transgenic RNAi Project (TRiP) at Harvard Medical School/Tsinghua University were screened (manuscript in preparation). Many factors affecting ISC maintenance, viability and proliferation/differentiation were identified from this screen. Among these factors, HS biosynthetic enzymes (including Sfl, Sgl and the EXT proteins) were identified as candidates. Only single or paired *esg*^+^ cells are observed in control flies (Fig. 1A). However, the number of *esg*^+^ cells was significantly increased when *sﬂ* was depleted in progenitors. *esg^+^* cells formed clusters and GFP was expressed in polyploid cells, indicative of midgut homeostasis loss (Fig. 1B,C,I). *sﬂ* encodes the only *Drosophila* HS N-deacetylase/N-sulfotransferase, which catalyzes the ﬁrst step of HS modiﬁcation (Esko and Lindahl, 2001; Lin, 2004). The RNAi off-target effect could be excluded as induction of two independent RNAi constructs against *sfl* produced a similar phenotype (Fig. 1B,C,I) and the knockdown efficacy of these RNAi lines was confirmed by qRT-PCR (Fig. S1). When *sgl* and EXT genes were knocked down, midgut homeostasis was also lost (Fig. 1D,I). Consistent with the increase of progenitors, we observed a significant increase of the number of cells undergoing mitosis in these intestines (Fig. 1J; Fig. S2). Meanwhile, many large *esg*^+^ cells expressed mature EC-marker PDM1, indicative of intestinal homeostasis loss (Fig. S3). Previous studies had demonstrated that mutations in these HS biosynthetic genes led to striking reductions in HS levels (Bornemann et al., 2004; Han et al., 2004; Takei et al., 2004; Toyoda et al., 2000a,b). Consistently, we found HS was effectively abrogated in *tub^ts^>botv^RNAi^* intestines (Fig. S4). These data demonstrate that HS in progenitors restricts ISC proliferation, thereby maintaining midgut homeostasis. As EXT3/Botv participates in the earliest steps of HS biosynthesis, we mainly focused on *botv* for further analysis.

### HS in progenitors negatively regulates ISC proliferation and differentiation

**Fig. 2.**
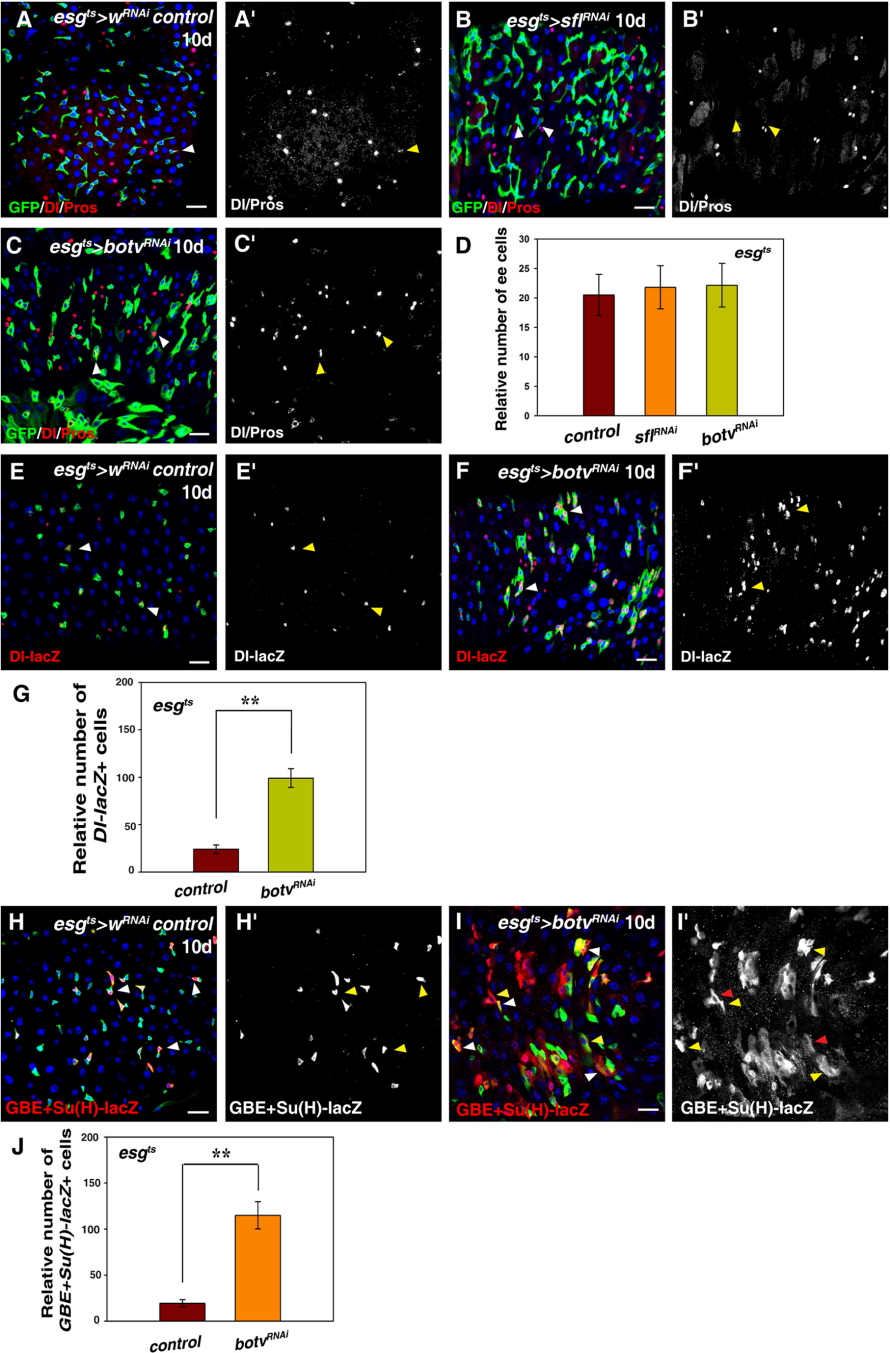
**HS in progenitors negatively regulates ISC proliferation and differentiation.** (A) Dl (ISC marker) and Pros (ee marker) (red) in control intestines (white arrowhead). Split channel for Dl and Pros (A′, in grayscale) in control intestines (yellow arrowhead). (B) Dl and Pros (red) in *esg^ts^>sfl^RNAi^* intestines (white arrowheads). Split channel for Dl and Pros (B′, in grayscale) in *esg^ts^>sfl^RNAi^* intestines (yellow arrowheads). (C) Dl and Pros (red) in *esg^ts^>botv^RNAi^* intestines (white arrowheads). Split channel for Dl and Pros (C′, in grayscale) in *esg^ts^>botv^RNAi^* intestines (yellow arrowheads). (D) Quantification of the relative number of ee cells in intestines with indicated phenotypes. *n*=10–15 intestines. mean±s.d. is shown. No obvious change in the number of ee cells is observed. (E) *Dl-lacZ* (red) in control intestines (white arrowheads). Split channel for *Dl-lacZ* (E′, in grayscale) in control intestines (yellow arrowheads). (F) *Dl-lacZ* (red) in *esg^ts^>botv^RNAi^* intestines (white arrowheads). Split channel for *Dl-lacZ* (F′, in grayscale) in *esg^ts^>botv^RNAi^* intestines (yellow arrowheads). (G) Quantification of the relative number of *Dl-lacZ*^+^ cells in control and *esg^ts^>botv^RNAi^* intestines. *n*=10–15 intestines. mean±s.d. is shown. **P<0.01. (H) EBs [by *GBE+Su(H)-lacZ* in red] in control intestines (white arrowheads). Split channel for *GBE+Su(H)-lacZ* (H′, in grayscale) in control intestines (yellow arrowheads). (I) The number of EBs [by *GBE+Su(H)-lacZ* in red] is dramatically increased in *esg^ts^>botv^RNAi^* intestines (white and yellow arrowheads). Split channel for *GBE+Su(H)-lacZ* (I′, in grayscale) in *esg^ts^>botv^RNAi^* intestines (yellow and red arrowheads). Note that the size of the larger *GBE+Su(H)-lacZ*^+^ cells is smaller compared to the size of the neighboring wild-type EC cells [polyploid *GBE+Su(H)-lacZ*^−^ cells], indicating that HS chains also affect EC maturation (yellow arrowheads in I). (J) Quantification of the relative number of *GBE+Su(H)-lacZ*^+^ cells in control and *esg^ts^>botv^RNAi^* intestines. *n*=10–15 intestines. mean±s.d. is shown. **P<0.01. GFP in green, blue indicates DAPI staining for DNA. Scale bars: 20 μm.

We examined the identity of these *esg*^+^ cells in the absence of HS. We found that the number of ISCs (by Dl and *Dl-lacZ*) in *esg^ts^>sfl^RNAi^* and *esg^ts^>botv^RNAi^* intestines was significantly increased compared to those in the control flies (Fig. 2A–C,E–F). The number of EBs [by *GBE+Su(H)-lacZ*] was also significantly increased in *esg^ts^>botv^RNAi^* intestines compared to those in the control flies (Fig. 2H–J). However, no obvious change in the number of ee cells (by Pros) was observed in these intestines (Fig. 2A–D). We found that the size of the large GFP^+^ cells (premature/mature ECs) was smaller than that of fully differentiated ECs, indicating that HS may also regulate EC maturation (Fig. 2H,I). We also observed a significant increase of ISCs when HS synthesis was disrupted in ISCs using ISC-specific driver *Dl^ts^* (data not shown). Taken together, these data demonstrate that HS in progenitors negatively regulates ISC proliferation and differentiation under physiological conditions.

Consistent with increased ISC proliferation, we found the number of *10xSTATGFP^+^* cells and the intensity of *10xSTATGFP* signal in *esg^ts^>botv^RNAi^* intestines were both significantly increased compared to those in the control flies (Fig. S5A–D). However, the expression of the JAK/STAT pathway cytokines (*Upd1-3*) was only mildly increased in *esg^ts^>botv^RNAi^* intestines compared to those in control intestines as determined by qRT-PCR (Fig. S6A). We examined whether the accumulation of *esg*^+^ cells observed in *esg^ts^>botv^RNAi^* intestines resulted from the expression of cytokines in progenitors. We found that neither individual nor simultaneous depletion of cytokines could suppress the accumulation of *esg*^+^ cells observed in those intestines (Fig. S6B–L). These data indicate that cytokines are unlikely produced in these *HS-deficient* progenitors.

### HSPGs (except Perlecan) may play redundant roles in progenitor proliferation

HSPGs comprise a core protein to which HS chains are attached. Our data demonstrate that HS in progenitors inhibits ISC proliferation. We explored which HSPG(s) are required for ISC proliferation. A previous study indicated that loss of the core protein of Perlecan (Per) caused detachment of ISC from basement membrane, resulting in loss of ISC proliferation (You et al., 2014). Dally and Dally-like (Dlp) are two major Glypicans in *Drosophila* (Esko and Lindahl, 2001; Esko and Selleck, 2002; Lin, 2004). We examined whether the other HSPGs, except Per, are required for ISC proliferation. We first examined the expression pattern of Dally and Dlp in the intestines. We found that Dally was mainly expressed in ECs (Fig. S7A,B), while Dlp was mainly expressed in the visceral muscles (VMs), and at low levels in the midgut epithelium (Fig. S7C,D). Effective RNAi constructs against *dally* and *dlp* were utilized to explore whether these HSPGs are required for ISC proliferation (Zhang et al., 2013). We found that neither individual nor combinational knockdown of *dally* and *dlp* affected ISC proliferation significantly (Fig. S7E–H). We also found that neither individual nor combinational knockdown of the other HSPGs, *sdc* and *cow*, as significantly affected ISC proliferation as those observed in HS-deficient progenitors (data not shown). These data indicate that these HSPGs (except Per) may play redundant roles in ISC proliferation. Therefore, we focused on HS chains, but not individual HSPGs, for further investigation.

### HS is required for Dpp signal activation in progenitors

**Fig. 3.**
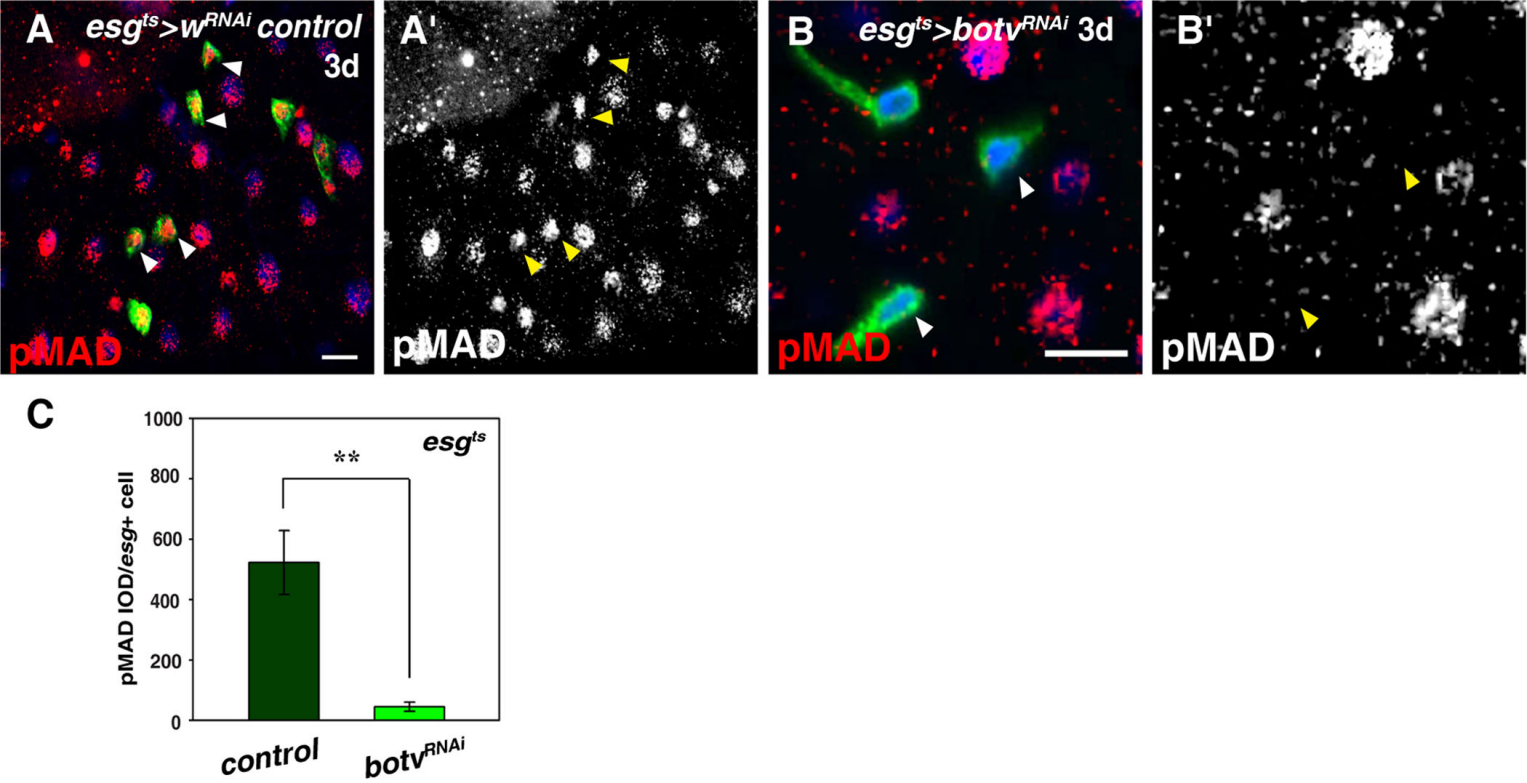
**Loss of HS leads to Dpp signal inactivation.** (A) Dpp signaling (by pMAD in red) in control intestines. Dpp signaling is highly activated in ECs and progenitors (green) (white arrowheads). Split channel for pMAD (A′, in grayscale) (yellow arrowheads). (B) Dpp signaling (by pMAD in red) in progenitors is eliminated in *esg^ts^>botv^RNAi^* intestines (white arrowheads). Split channel for pMAD (B′, in grayscale) (yellow arrowheads). (C) Quantification of by pMAD signal after knockdown of *botv* in progenitors. IOD was used. *n*≥4. mean±s.d. is shown. **P<0.01. In all panels except graphs, GFP is in green, blue indicates DAPI staining for DNA. Scale bars: 20 μm.

The above mentioned experiments demonstrate that HS in progenitors is required for midgut homeostasis under normal conditions. We explored the underlying mechanism of how HS controls midgut homeostasis. HS is required for the activation of many signaling pathways, including Wg, JAK/STAT, Notch and BMP (Belenkaya et al., 2004; Bellaiche et al., 1998; Binari et al., 1997; Bornemann et al., 2004; Dani et al., 2012; Filmus et al., 2008; Fujise et al., 2003; Jackson et al., 1997; Kamimura et al., 2006; Levings and Nakato, 2017; Lin and Perrimon, 1999, 2000, 2002; Liu et al., 2010; Mii et al., 2017; Takei et al., 2004; Yan and Lin, 2009; Yu et al., 2017; Zhang et al., 2013). Interestingly, the majority of these signaling pathways positively regulate ISC proliferation and differentiation, while Dpp signaling negatively regulates ISC proliferation and differentiation (Guo et al., 2013; Jiang and Edgar, 2009; Jiang et al., 2011, 2009; Li et al., 2013a,b, 2014; Lin et al., 2008; Tian and Jiang, 2014; Tian et al., 2015; Zhou et al., 2015). Notch signaling is not blocked upon loss of HS as the number of *GBE+Su(H)-lacZ*^+^ cells was dramatically increased, indicating that Notch signaling is not affected by HS depletion (Fig. 2H–J). Therefore, we speculated that HS may be required for Dpp signal activation in progenitors. To confirm our hypothesis, we examined Dpp signal activation in the absence of HS. Dpp signaling (by pMAD) is mainly activated in ECs, but also in progenitors in control flies (Fig. 3A) (Li et al., 2013b; Tian and Jiang, 2014; Zhou et al., 2015). When HS was depleted in progenitors, pMAD signal in progenitors was abolished, supporting the notion that HS is required for Dpp signal activation in progenitors (Fig. 3A–C).

### Dpp signaling negatively regulates ISC proliferation and midgut homeostasis under physiological conditions

**Fig. 4.**
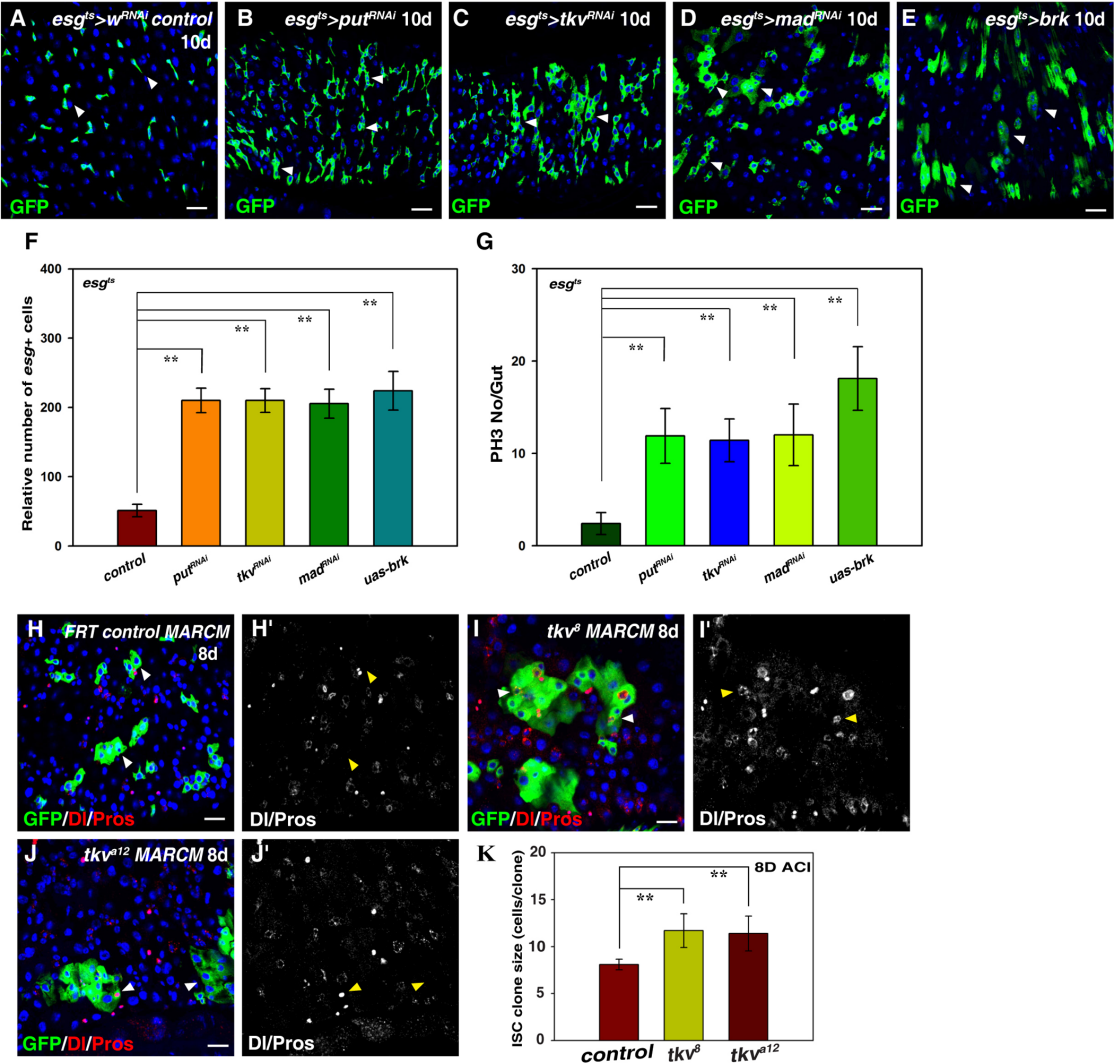
**Dpp signaling in ISCs restricts ISC proliferation.** (A) *esg*^+^ cells (green) in control flies at 29°C for 10 days (white arrowheads). (B) The number of *esg*^+^ cells (green) is dramatically increased in *esg^ts^>put^RNAi^* flies at 29°C for 10 days (white arrowheads). (C) The number of *esg*^+^ cells (green) is dramatically increased in *esg^ts^>tkv^RNAi^* flies at 29°C for 10 days (white arrowheads). (D) The number of *esg*^+^ cells (green) is dramatically increased in *esg^ts^>mad^RNAi^* flies at 29°C for 10 days (white arrowheads). (E) The number of *esg*^+^ cells (green) is dramatically increased in *esg^ts^>brk* flies at 29°C for 10 days (white arrowheads). (F) Quantification of the relative number of *esg^+^* cells in different genotypes indicated. mean±s.d. is shown. *n*=10–15 intestines. **P<0.01. (G) Quantification of the number of pH3/gut in different genotypes indicated. mean±s.d. is shown. *n*=10–15 intestines. **P<0.01. (H) ISC MARCM clones (green) in *FRT* control (8 days at 25°C, 8D ACI) (white arrowheads). Dl and Pros in red. Split channel for Dl and Pros (H′, in grayscale) (yellow arrowheads). (I,J) The size of *tkv^8^* (I) and *tkv^a12^* (J) ISC MARCM clones (green) is significantly increased (8D ACI) (white arrowheads). Dl and Pros in red. Split channel for Dl and Pros (I′,J′, in grayscale) (yellow arrowheads). (K) Quantiﬁcation of ISC clone size in control and *tkv* mutants (8D ACI). Note that the size of *tkv* mutant ECs is smaller than neighboring wild-type ECs. mean±s.d. is shown. *n*=10. **P<0.01. In all panels except graphs, GFP is in green, blue indicates DAPI staining for DNA. Scale bars: 20 μm. Note that images for the control flies in A are reproduced from Fig. 1.

We then explored whether the defects observed in HS-depletion intestines are direct consequences of Dpp signal inactivation. We first depleted the expression of several key components of the Dpp signaling pathway, including the type II receptor Punt (Put), the type I receptor Thickveins (Tkv) and Mother against Dpp (Mad), in the progenitors using functional RNAi constructs by the *esg^ts^* driver (Li et al., 2013b; Xu et al., 2018). We found that the number of *esg^+^* cells was dramatically increased in these intestines and many polyploid cells expressed GFP, indicative of midgut homeostasis loss (Fig. 4A–D,F). The observed phenotypes are almost identical to those of HS depletion. We further ectopically expressed *brinker* (*brk*) in progenitors to block Dpp signaling (Jaźwińska et al., 1999). Similarly, the number of *esg^+^* cells was dramatically increased in *esg^ts^>brk* intestines, and *esg^+^* cells formed clusters with many polyploid cells expressing GFP (Fig. 4E,F). Consistently, we observed a significant increase of the number of pH3^+^ cells in these intestines (Fig. 4F; Fig. S8). These data show that Dpp signaling negatively regulates ISC proliferation and midgut homeostasis under physiological conditions.

We examined the identity of the *esg*^+^ cells upon Dpp signaling inactivation in progenitors. We found that the number of ISCs (by Dl and *Dl-lacZ*) in *esg^ts^>tkv^RNAi^* intestines was significantly increased compared to those in the control flies (Fig. S9A,B, and data not shown). No obvious change in the number of ee cells was observed in these intestines (Fig. S9A,B). We found the number of EBs [by *GBE+Su(H)-lacZ*] was also significantly increased in *esg^ts^>put^RNAi^* and *esg^ts^>tkv^RNAi^* intestines compared to those in the control flies (Fig. S9C–F). Consistently, we found that the size of the polyploid GFP^+^ cells was smaller than fully differentiated ECs, indicating that Dpp signaling may also regulate EC maturation (Fig. S9C–E). Furthermore, we generated *tkv* ISC clones using the MARCM technique (Lee and Luo, 2001). We utilized two amorphic *tkv* mutants, *tkv^8^* and *tkv^a12^*. The ISC clone size of both *tkv* mutants was significantly increased compared with control clones (Fig. 4H–K). Together, these data demonstrate that Dpp signaling in progenitors negatively regulates ISC proliferation and differentiation to maintain midgut homeostasis under normal conditions.

**Fig. 5.**
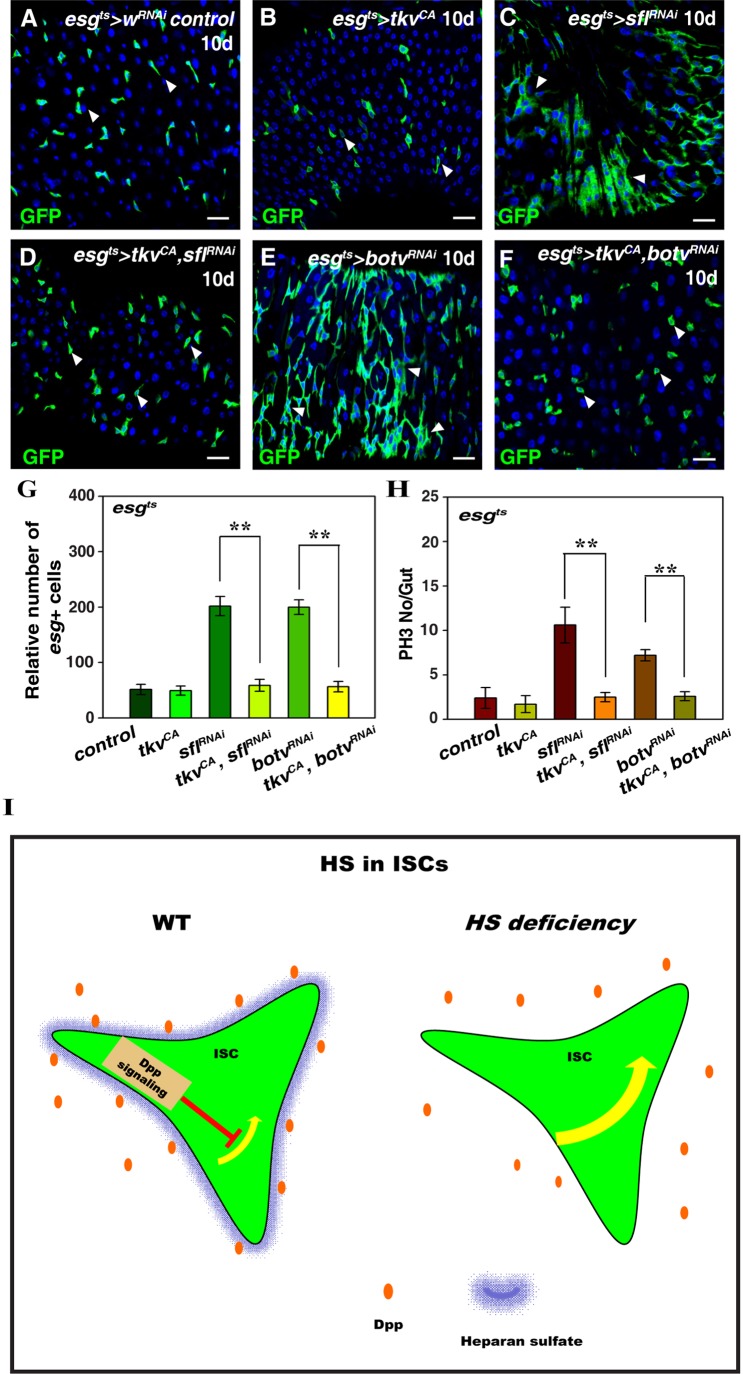
**Ectopic activation of Dpp signaling completely rescued defects observed in the absence of HS.** (A) *esg*^+^ cells (green) in control flies at 29°C for 10 days (white arrowheads). (B) Progenitors (ISC+EBs, green) in *esg^ts^>tkv^CA^* intestines (white arrowheads). (C) The number of *esg*^+^ cells (green) is dramatically increased in *esg^ts^>sfl^RNAi^* flies at 29°C for 10 days (white arrowheads). (D) Expression of *tkv^CA^* could completely rescue increased ISC proliferation observed in *esg^ts^>sfl^RNAi^* intestines (white arrowheads). (E) The number of *esg*^+^ cells (green) is dramatically increased in *esg^ts^>botv^RNAi^* flies at 29°C for 10 days (white arrowheads). (F) Expression of *tkv^CA^* could completely rescue increased ISC proliferation observed in *esg^ts^>botv^RNAi^* intestines (white arrowheads). (G) Quantification of the relative number of *esg^+^* cells in different genotypes indicated. mean±s.d. is shown. *n*=10–15 intestines. **P<0.01. (H) Quantification of the number of pH3 per gut in different genotypes indicated. mean±s.d. is shown. *n*=10–15 intestines. **P<0.01. (I) Model of HS function in progenitor cells. In all panels except graphs, GFP is in green, blue indicates DAPI staining for DNA. Scale bars: 20 μm. Note that images for the control flies in A,C and E are reproduced from Fig. 1.

To further confirm that Dpp signaling inactivation is the direct consequence of HS disruption, we performed rescue experiments. Our rationale is: if Dpp signal inactivation is the direct consequence of HS disruption, then restoring Dpp signaling in *HS-depleted* intestines will completely rescue the defects observed in *HS-deficient* intestines. Interestingly, co-expression of a constitutively active form of *tkv* (*tkv^CA^*) with either *sfl^RNAi^* or *botv^RNAi^* completely rescued the defects observed in *esg^ts^>sfl^RNAi^* and *esg^ts^>botv^RNAi^* intestines respectively (Fig. 5A–G). Moreover, the increased number of ISCs undergoing mitosis was also completely rescued in these intestines (Fig. 5H; Fig. S10). These data indicate that Dpp signal inactivation is very likely the direct consequence of HS depletion. Taken together, these data show that HS is required for Dpp signaling in progenitors to maintain midgut homeostasis under physiological conditions (Fig. 5I).

## DISCUSSION

Residential stem cells must respond to extrinsic signals to properly adjust their proliferation and differentiation rate to maintain tissue homeostasis under normal conditions. However, how extrinsic signals are transduced into ISCs is poorly understood. Here we reveal that HS is required for Dpp signaling in progenitors to maintain midgut homeostasis under normal conditions.

HSPGs are involved in multiple biological processes, including cell proliferation/differentiation, cell adhesion, extracellular matrix assembly and growth factor diffusion/storage (Esko and Selleck, 2002; Gomes et al., 2013; Iozzo, 1998; Kamimura et al., 2011; Lin, 2004; Lin and Perrimon, 2000; Bernfield et al., 1999; Sarrazin et al., 2011; Yan et al., 2010). The highly diversiﬁed functions of HSPGs are mediated by the varied nature of the core proteins and speciﬁc HS modiﬁcations (Esko and Lindahl, 2001; Esko and Selleck, 2002; Lin, 2004). Although HS plays important roles in the diversified functions of HSPGs, the role of HS in ISC proliferation and differentiation under normal homeostasis has not been systemically explored (Guo et al., 2014; Takemura and Nakato, 2017; You et al., 2014). Our data demonstrate that HS in progenitors negatively regulates ISC proliferation and differentiation to maintain midgut homeostasis under normal conditions (Fig. 1). Consistent with our results, a previous study revealed that heparan sulfate 3-O sulfotransferases (Hs3sts) negatively regulate ISC proliferation (Guo et al., 2014). The extracellular endosulfatases, Sulfs (Sulf1 in *Drosophila*), specifically remove *6-O* sulfate groups (at the *6-O* position of glucosamine residues) from highly sulfated regions of HS. Interestingly, a recent study found that ISC proliferation was increased in the absence of *sulf1* (Takemura and Nakato, 2017). These data indicate that the levels of HS need to be properly controlled for adequate ISC proliferation.

How does HS restrict ISC proliferation? Our data support the notion that under normal conditions, HS is required for Dpp signal activation, which in turn negatively regulates ISC proliferation and differentiation to maintain midgut homeostasis based on the following observations: (1) Dpp signaling was greatly diminished in the absence of HS in ISCs (Figs 1 and 2); (2) Dpp signaling inactivation in progenitors led to increased ISC proliferation and midgut homeostasis loss under normal conditions (Fig. 3); and (3) most importantly, restoring Dpp signaling in the absence of HS in progenitors completely rescued increased ISC proliferation and tissue homeostasis loss (Fig. 5). Although we cannot exclude the possibility that HS may be required for activating other signaling pathways in progenitors, our data favor the notion that HS mainly activates Dpp signaling in progenitors, regardless of the sources of Dpp ligand. Previous studies showed that injury-induced BMP signaling negatively regulates midgut homeostasis; our results indicate that under physiological conditions, Dpp signaling also negatively regulates ISC proliferation and differentiation to maintain tissue homeostasis (Guo et al., 2013; Tian et al., 2017; Zhou et al., 2015). Consistent with previous studies, our results show that Dpp signaling negatively regulates ISC proliferation and differentiation (Guo et al., 2013; Zhou et al., 2015). However, Guo et al.’s, Zhou et al's and our data are contradictory to Tian et al's findings (Tian et al., 2017). We speculate that the paradox may be resulted from the differences in genetic backgrounds, the drivers used and the experimental conditions.

Interestingly, we found that co-expression of *dpp* could completely rescue the defects observed in *esg^ts^>botv^RNAi^* intestines, while co-expression of *gbbGFP* could only partially rescue the defects observed in *esg^ts^>botv^RNAi^* intestines, indicating that Dpp may be more potent than Gbb (Fig. S11). Our finding that HS is required for Dpp signal activation is not unique for ISCs. Previous studies showed that both *sfl* and *dally* are required for Dpp signal activation to control germline stem cell (GSC) maintenance in ovary (Guo and Wang, 2009; Hayashi et al., 2009). Therefore, regardless of the sources of Dpp molecules, we reveal that HS is required for Dpp signal activation to maintain midgut homeostasis under normal conditions.

## MATERIALS AND METHODS

### Fly lines and cultures

Flies were maintained on standard media at 25°C. Crosses were raised at 18°C in humidity controlled incubators, or as otherwise noted. Flies hatched in 18°C incubators (2–3 days old) were picked and transferred to 29°C incubator, unless otherwise specified. Flies were transferred to new vials with fresh food every day, and dissected at time points specified in the text. In all experiments, only the female posterior midgut was analyzed. Information for alleles and transgenes used in this study can be found either in FlyBase or as noted: *esgGal4, UAS-GFP, tubGal80^ts^* (*esg^ts^*, gift from N. Perrimon, Harvard University), *esgGal4, UAS-RFP, tubGal80^ts^, sfl^RNAi^* (BL34601, BL50538), *tubGal80^ts^, tubGal4* (*tub^ts^*)*, sgl^RNAi^* (BL65348), *ttv^RNAi^* (BL51480), *sotv^RNAi^* (BL52883), *botv^RNAi^* (GD2083, BL61257), *dally^RNAi^* (BL33952), *dlp^RNAi^* (BL34089, BL34091), *Upd1^RNAi^* (BL33680, BL28722), *Upd2^RNAi^* (BL33949, BL33988), *Upd3^RNAi^* (BL32859, BL28575), *GBE+Su(H)-lacZ* (gift from S. Bray, University of Cambridge) (Furriols and Bray, 2001), *10XSTATGFP* (gift from G. Baeg, National University of Singapore) (Bach et al., 2007), *esg-lacZ^B7–2-22^, tubGal4^ts^*, *Dl^05151^* (*Dl-lacZ*), *tkv^RNAi^* (VDRC3059, NIG 14026R-1, and HMS02185), *put^RNAi^* (GL00069, HMS01944 and NIG 7904R-2), *mad^RNAi^* (GL01527 and GLV21013), *w(white)^RNAi^* (BL33623) and/or *Gal4^RNAi^* (HMS504, from TRiP at Harvard Medical School) were used as control, *FRT40A-tkv^8^*, *FRT40A-tkv^a12^, UAS-brk, UAS-tkv^Q253D^* (*tkv^CA^*)*, dally^P1^* (Nakato et al., 1995), *dally^06464^* (*dally^P2^,* BL11685). *hsFlp, ActGal4, UAS-GFP*; *FRT40A-tubGal80* (for MARCM clonal analysis), *UAS-dpp*, *UAS-gbb-GFP* (BL63507 and BL63508)*.*

### RNAi knockdown and overexpression experiments

To address gene function in ISCs, *esgGal4, UAS-GFP, tubGal80^ts^* (*esg^ts^*) was used, and crosses (unless stated otherwise) were maintained at 18°C to bypass potential requirements during early developmental stages. 2–3 days old progeny with the desired genotypes were collected after eclosion and maintained at 29°C to inactivate Gal80^ts^ before dissection and immunostaining. The flies were transferred to new vials with fresh food every day. Both *UAS-dsRNA* and *UAS-shRNA* transgene stocks were used in this study. If possible, several dsRNA or shRNA lines were tested for each gene (the lines listed above showed similar phenotypes), and one or two RNAi lines were used for detailed study. To detect JAK/STAT signaling, *esgGal4, UAS-RFP; 10XSTATGFP*, *tubGal80^ts^* driver was used. The time points that the flies are analyzed/dissected were indicated in the text.

### Immunostainings and fluorescence microscopy

For standard immunostaining, intestines were dissected in 1× PBS (10 mM NaH_2_PO_4_/Na_2_HPO_4_, 175 mM NaCl, pH 7.4), and fixed in 4% paraformaldehyde for 25 min at room temperature. Samples were rinsed, washed with 1× PBT (0.1% Triton X-100 in 1× PBS) and blocked in 5% horse serum in 1× PBT for 45 min. Embryos were ﬁxed and stained following standard protocol. Primary antibodies were added to the samples and incubated at 4°C overnight. The following primary antibodies were used: mouse mAb anti-Dl [C594.9B, 1:50, developed by S. Artavanis-Tsakonas, Developmental Studies Hybridoma Bank (DSHB)], mouse mAb anti-Prospero (MR1A, 1:100, developed by C.Q. Doe, DSHB), mouse mAb anti-Dlp (13G8, 1:100, developed by P. A. Beachy, DSHB), rabbit mAb anti-pMAD3 (Epitomics, 1:200), rabbit anti-β-glactosidase (Cappel, 1:5000), mouse anti-β-glactosidase (Cell Signaling, 1:1000), rabbit anti-PDM1 (gift from Xaohang Yang, Zhejiang University, 1:1000), rabbit anti-pH3 (pSer10, Millipore, 1:2000, USA) and mouse anti-HS (clone F58-10E4 and F69-3G10, 1:100, Amsbio). For 3G10 staining, fixed intestines were pretreated by heparanase III (2 U/ml, Sigma-Aldrich) at 37°C for 2 h to expose the neo-epitope site. The primary antibodies were detected by fluorescent-conjugated secondary antibodies from Jackson ImmunoResearch Laboratories. Secondary antibodies were incubated for 2 h at room temperature. DAPI (Sigma-Aldrich, 0.1 μg/ml) was added after secondary antibody staining. The samples were mounted in mounting medium (70% glycerol containing 2.5% DABCO). All images were captured by a Zeiss LSM780 inverted confocal microscope, and were processed in Adobe Photoshop and Illustrator.

### MARCM ISC clone analysis

The clonal analyses were achieved using the MARCM system. The ISC clones were induced by heat shocking 3–5 day-old adult flies at 37°C for 60 min. The flies were maintained at 25°C incubator and transferred to new vials with fresh food every day. The sizes of the marked clones were assayed at 8 days after clone induction (8D ACI, clones from at least 10 midguts for each genotype were assayed).

### Signal quantiﬁcation

Image-Pro-Plus 6.0 software was used for pMAD signal quantiﬁcation. Two parameters, integrated optical density (IOD) and area, were used in the analysis. For pMAD signal quantification in ISCs, a pixel ﬁlter was set to ensure that the area of interest did not include objects larger than 20 pixels, excluding *esg^−^* cells (which are ECs and ee cells). IOD value per ISC was used. At least four different images were analyzed for each sample.

### qRT-PCR

RNA was extracted from 30 flies or guts using TRIzol (Invitrogen). RNA was cleaned using RNAeasy (QIAGEN), and complementary DNA (cDNA) was synthesized using the iScript cDNA synthesis kit (Bio-Rad). Quantitative PCR was performed using the iScript one-step RT-PCR SYBR green kit (Bio-Rad). Data were acquired using an iQ5 System (Bio-Rad). qRT-PCR was performed in duplicate on each of three independent biological replicates. All results are presented as mean±s.d. of the biological replicates. The ribosomal gene *RpL11* was used as the normalization control. Primers used for qRT-PCR can be found in Table S1.

### Data analysis

pH3 numbers were scored manually under Zeiss Imager Z2/LSM780 microscope for indicated genotypes. To determine the relative number of *esg*^+^ cells per confocal image (including *esg>GFP*^+^, *esg-lacZ*^+^ and *10xSTATGFP*^+^), confocal images of 40× lens/1.0 zoom from a defined posterior midgut region (R4-R5 regions) of different genotypes indicated were acquired. The relative number of *esg*^+^ and *GBE+Su(H)-lacZ*^+^ cells was determined using Image-Pro Plus software from each confocal image. The number of intestines scored is indicated in the text. Fluorescence intensity of *10xSTATGFP* and HS was measured using Image Pro Plus 6.0 (measure/count function). The data are presented as the means±standard deviation and two-tailed Student's *t*-tests were performed for statistical comparisons. PEMS 3.1 software was used for s.d. analyses and Sigma Plot software for graph generation. *P<0.05; **P<0.01. The graphs were further modified using Adobe Photoshop and Illustrator.

## Supplementary Material

Supplementary information
