# Supplementary material for: Heparan sulfate negatively regulates intestinal stem cell proliferation in Drosophila adult midgut
Source: Biol Open. 2019 Oct 15;8(10):bio047126. doi: 10.1242/bio.047126 (PMC6826283; doi:10.1242/bio.047126)
Supplement: Supplementary information [file biolopen-8-047126-s1.pdf]

Supplementary material

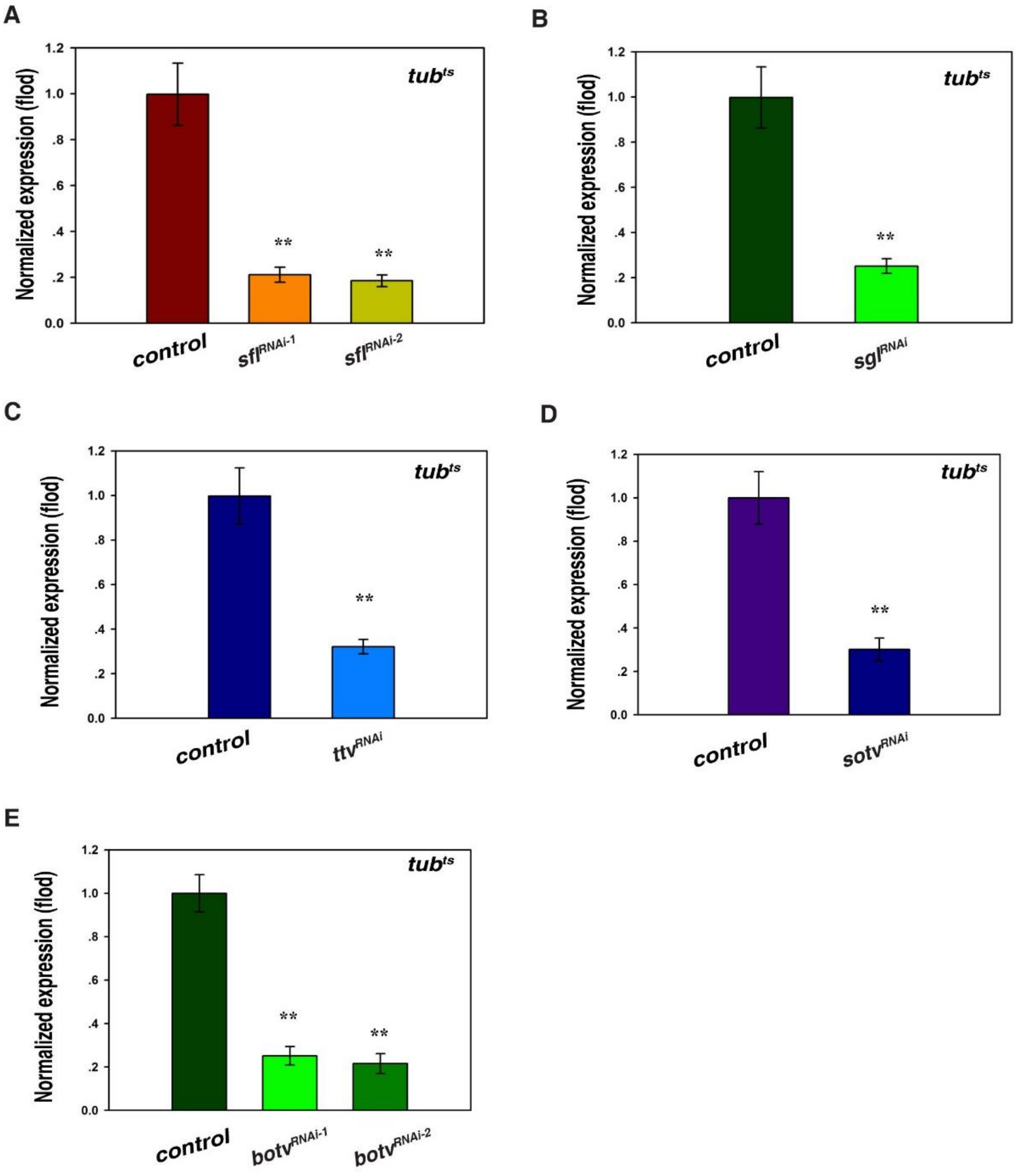

Biology Open • Supplementary information

Fig.S1. Knockdown efficacy of RNAi lines against genes encoding enzymes in HS chain biogenesis. (A) Knockdown efficacy of two different *sfl* RNAi lines used in this study was determined by qRT-PCR quantification from tub^{ts}>sfl^{RNAi} flies (ovaries were not included). Ribosomal gene *RpL11* was used as normalization control. means ± SD are shown. ***p* < 0.01. (B) Knockdown efficacy of *sgl* RNAi line used in this study was determined by qRT-PCR quantification from tub^{ts}>sgl^{RNAi} flies (ovaries were not included). Ribosomal gene *RpL11* was used as normalization control. means ± SD are shown. ***p* < 0.01. (C) Knockdown efficacy of *ttv* RNAi line used in this study was determined by qRT-PCR quantification from tub^{ts}>ttv^{RNAi} flies (ovaries were not included). Ribosomal gene *RpL11* was used as normalization control. means ± SD are shown. ***p* < 0.01. (D) Knockdown efficacy of *sotv* RNAi line used in this study was determined by qRT-PCR quantification from tub^{ts}>$sotv^{RNAi}$ flies (ovaries were not included). Ribosomal gene *RpL11* was used as normalization control. means ± SD are shown. ***p* < 0.01. (E) Knockdown efficacy of two different *botv* RNAi lines used in this study was determined by qRT-PCR quantification from tub^{ts}>$botv^{RNAi}$ flies (ovaries were not included). Ribosomal gene *RpL11* was used as normalization control. means ± SD are shown. ***p* < 0.01.

Biology Open • Supplementary information

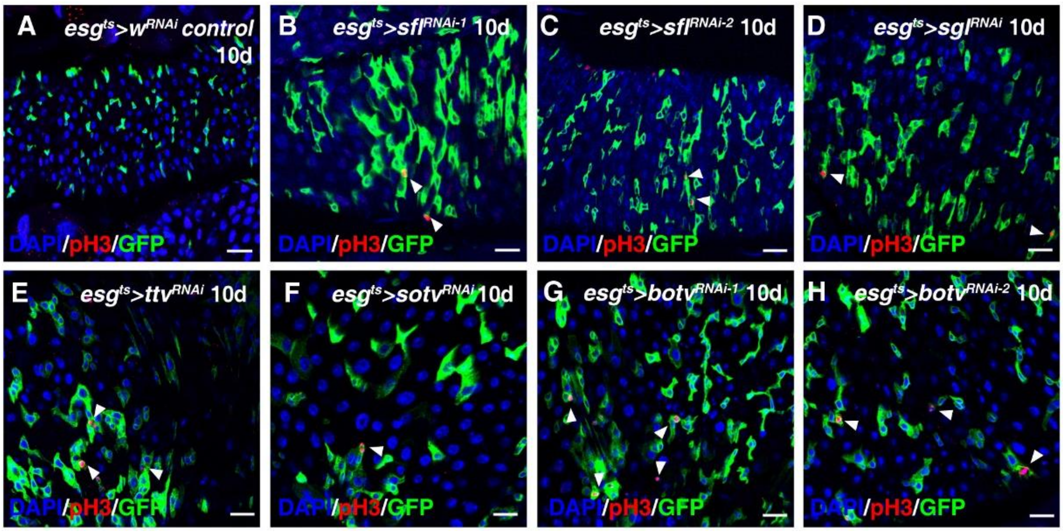

Fig. S2. ISC proliferation is increased upon loss of HS in progenitors. (A) pH3 (red) in control intestines. (B) pH3 (red) in *esg*^{ts}>*sfl*$^{RNAi\text{-}1}$ intestines (white arrowheads). (**C**) pH3 (red) in *esg*^{ts}>*sfl*$^{RNAi\text{-}2}$ intestines (white arrowheads). (D) pH3 (red) in *esg*^{ts}>*sgl*^{RNAi} intestines (white arrowheads). (E) pH3 (red) in *esg*^{ts}>*ttv*^{RNAi} intestines (white arrowheads). (F) pH3 (red) in *esg*^{ts}>*sotv*^{RNAi} intestines (white arrowhead). (G) pH3 (red) in *esg*^{ts}>*botv*$^{RNAi\text{-}1}$ intestines (white arrowheads). (H) pH3 (red) in *esg*^{ts}>*botv*$^{RNAi\text{-}2}$ intestines (white arrowheads). GFP in green, blue indicates DAPI staining for DNA. Scale bars: 20 µm. Please refer to Fig. 1J for quantification data.

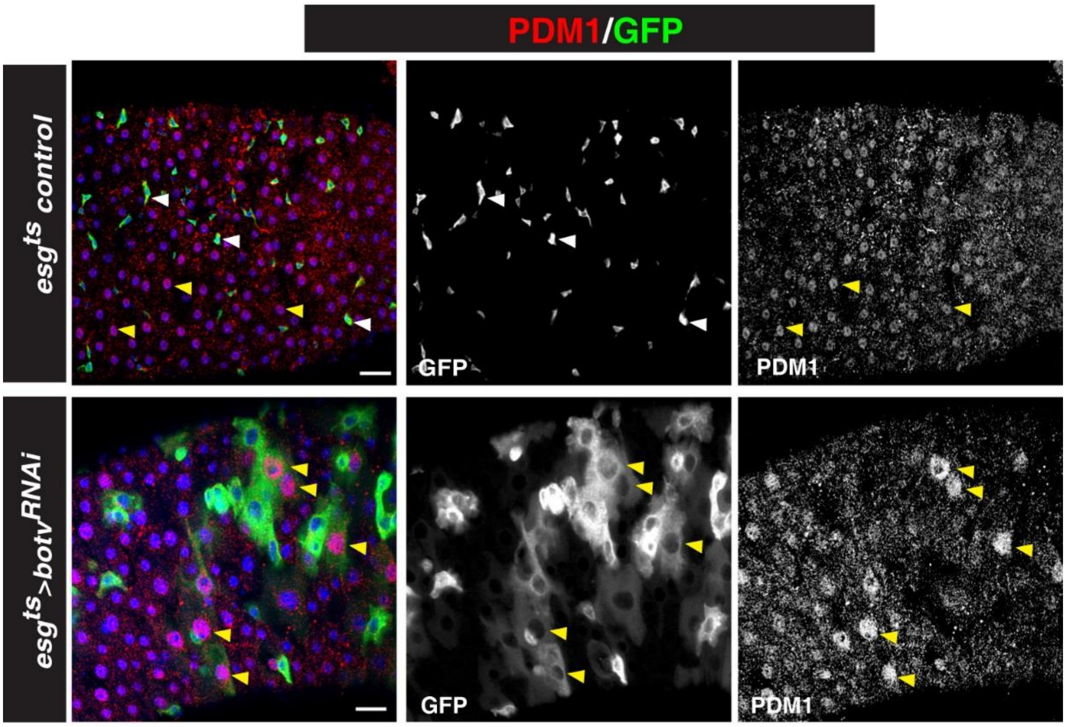

Fig. S3. Depletion of HS in progenitors results in intestinal homeostasis loss. (Upper panel) progenitors (green) and mature ECs (by PDM1, red) in control intestines (white and yellow arrowheads). (Lower panel) Many large *esg*$^{+}$ cells express mature EC-marker PDM1 (red) in *esg*^{ts}>*botv*^{RNAi} intestines (yellow arrowheads). Split channel for GFP and PDM1 was shown. GFP in green, blue indicates DAPI staining for DNA. Scale bars: 20 µm.

Biology Open • Supplementary information

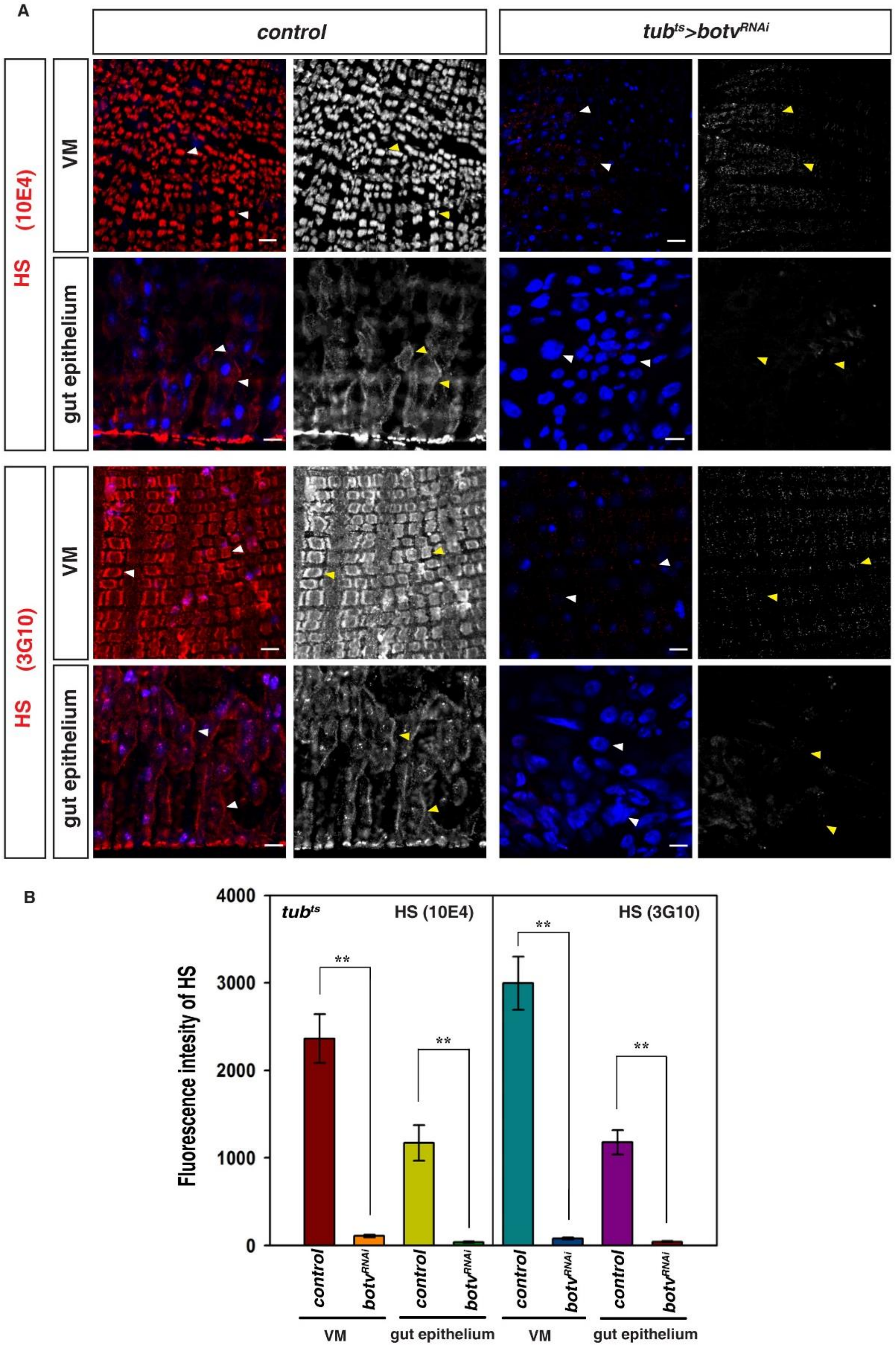

Biology Open • Supplementary information

Fig. S4. HS is abrogated in *tub^{ts}>botv^{RNAi}* intestines. (A) Immunofluorescent staining for HS (by 10E4 and 3G10 in red) in the visceral muscles (VMs) and the gut epithelium of control intestines, respectively (white and yellow arrowheads, left panels). *botv* deletion abrogated HS staining (both by 10E4 and 3G10) in the VMs and the gut epithelium (white and yellow arrowheads, right panels). Split channel for HS (in black-white) was shown (yellow arrowheads). (B) Quantification of mean fluorescent intensity of HS (by10E4 and 3G10) in control and *tub^{ts}>botv^{RNAi}* intestines. $n \geqslant 4$. means ± SD is shown. $^{**}p < 0.01$. Blue indicates DAPI staining for DNA (except graphs). Scale bars: 10 μm.

Biology Open • Supplementary information

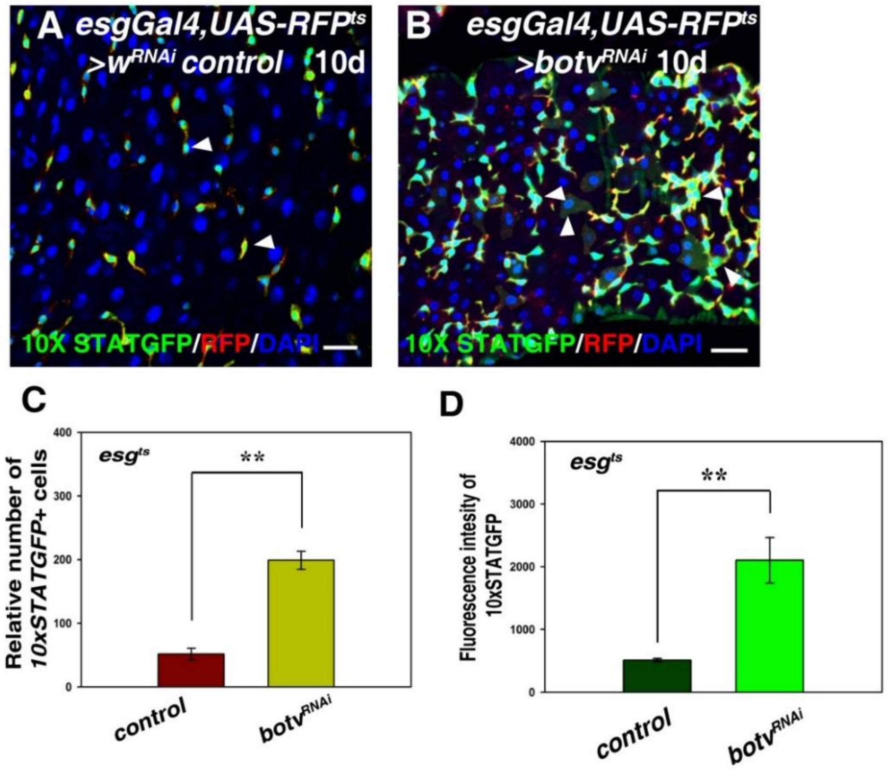

Fig. S5. JAK/STAT signaling is activated upon HS depletion in progenitors. (A) JAK/STAT signaling (by *10xSTATGFP*, green) in control intestines (*esgGal4^{ts}>RFP* to label progenitors in red) (white arrowheads). Note that *10xSTATGFP* is only activated in progenitor cells in the intestinal epithelium of control. (B) JAK/STAT signaling (by *10xSTAT-GFP*, green) is highly activated in *esg^{ts}>botv^{RNAi}* intestines (white arrowheads). (C) Quantification of the relative number of *10xSTATGFP^{+}* cells in control and *esg^{ts}>botv^{RNAi}* intestines. n = 10-15 testes. mean ± SD is shown. **p< 0.01. Please note that accurate quantification of the number of *10xSTATGFP^{+}* cells in *esg^{ts}>sfl^{RNAi}* and *esg^{ts}>botv^{RNAi}* intestines is not feasible as midgut homeostasis is lost in these intestines. (D) Quantification of fluorescence intensity of *10xSTATGFP^{+}* in control and *esg^{ts}>botv^{RNAi}* intestines. n = 10. mean ± SD is shown. **p< 0.01. Blue indicates DAPI staining for DNA. Scale bars: 20 μm.

Biology Open • Supplementary information

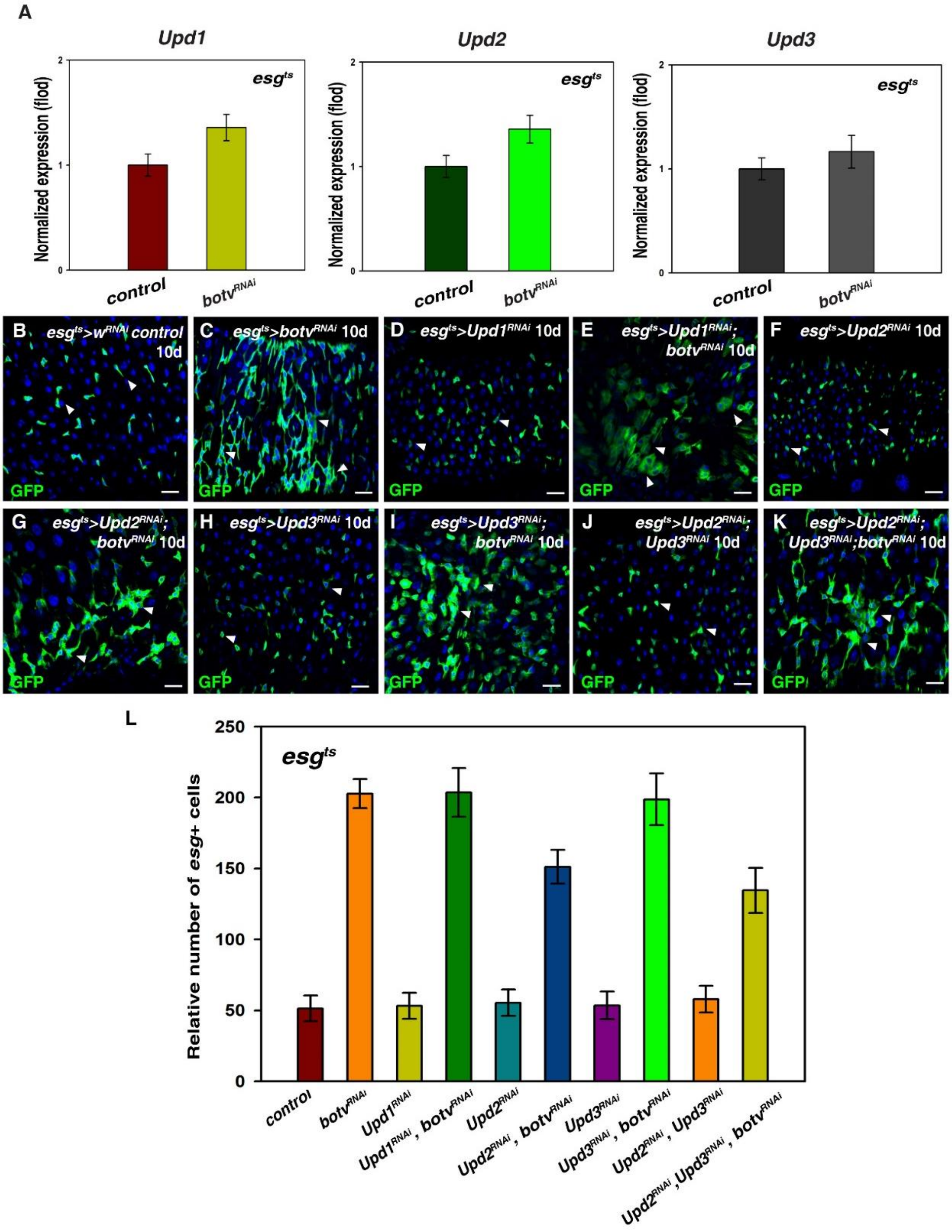

Fig. S6. Upds are unlikely produced in *HS-deficient* progenitors. (A) qRT-PCR quantification of *Drosophila* cytokines (*Upd1*, *Upd2*, and *Upd3*) mRNA expression from *esg*^{ts}>*botv*^{RNAi} whole midguts at 29°C for 10 days. Ribosomal gene *RpL11* was used as normalization control. mean ± SD are shown. (B) *esg*$^{+}$ cells (green) in control flies at 29°C for 10 days (white arrowheads). (C) The number of *esg*$^{+}$ cells (green) is

Biology Open • Supplementary information

dramatically increased in *esg*^{ts}>*botv*^{RNAi} flies at 29°C for 10 days (white arrowheads). (D) *esg*$^{+}$ cells (green) in *esg*^{ts}>*Upd1*^{RNAi} flies at 29°C for 10 days (white arrowheads). (E) No significant change in the number of *esg*$^{+}$ cells (green) is observed in *esg*^{ts}> *Upd1*^{RNAi}, *botv*^{RNAi} flies compared to those in *esg*^{ts}>*botv*^{RNAi} flies at 29°C for 10 days (white arrowheads). (F) *esg*$^{+}$ cells (green) in *esg*^{ts}>*Upd2*^{RNAi} flies at 29°C for 10 days (white arrowheads). (G) No significant change in the number of *esg*$^{+}$ cells (green) is observed in *esg*^{ts}> *Upd2*^{RNAi}, *botv*^{RNAi} flies compared to those in *esg*^{ts}>*botv*^{RNAi} flies at 29°C for 10 days (white arrowheads). (H) *esg*$^{+}$ cells (green) in *esg*^{ts}>*Upd3*^{RNAi} flies at 29°C for 10 days (white arrowheads). (I) No significant change in the number of *esg*$^{+}$ cells (green) is observed in *esg*^{ts}> *Upd3*^{RNAi}, *botv*^{RNAi} flies compared to those in *esg*^{ts}>*botv*^{RNAi} flies at 29°C for 10 days (white arrowheads). (J) *esg*$^{+}$ cells (green) in *esg*^{ts}>*Upd2*^{RNAi}, *Upd3*^{RNAi} flies at 29°C for 10 days (white arrowheads). (K) No significant change in the number of *esg*$^{+}$ cells (green) is observed in *esg*^{ts}> *Upd2*^{RNAi}, *Upd3*^{RNAi}, *botv*^{RNAi} flies compared to those in *esg*^{ts}>*botv*^{RNAi} flies at 29°C for 10 days (white arrowheads). (L) Quantification of the relative number of *esg*$^{+}$ cells in intestines with indicated phenotypes. n = 10-15 intestines. mean ± SD is shown. Blue indicates DAPI staining for DNA. Scale bars: 20 μm.

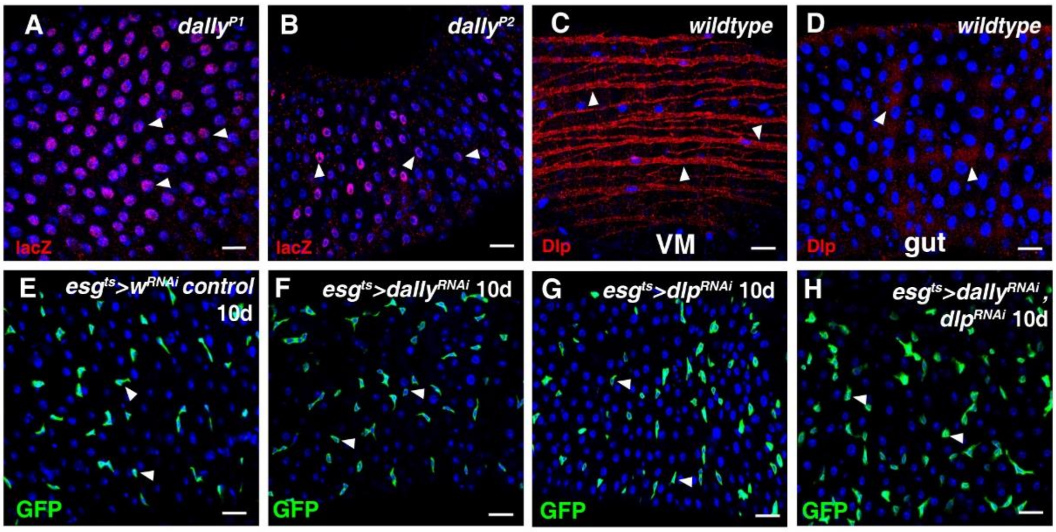

Fig. S7. HSPGs (except Per) are likely redundant for ISC proliferation. (A and B) Expression pattern of *dally* (by *dally*^{P1} (A) and *dally*^{P2} (B) in red) in intestines. *dally* (by *dally-lacZ*) is mainly expressed in ECs (white arrowheads). (C and D) Expression pattern of Dlp (red) in intestines. High levels of Dlp can be detected in the visceral muscles (VMs) and low levels in the intestinal epithelium (white arrowheads). (E) *esg*$^{+}$ cells (green) in control intestines at 29°C for 10 days (white arrowheads). (F) *esg*$^{+}$ cells (green) in *esg*^{ts}>*dally*^{RNAi} intestines at 29°C for 10 days (white arrowheads). (G) *esg*$^{+}$ cells (green) in *esg*^{ts}>*dlp*^{RNAi} intestines at 29°C for 10 days (white arrowheads). (H) *esg*$^{+}$ cells (green) in *esg*^{ts}>*dally*^{RNAi}, *dlp*^{RNAi} intestines at 29°C for 10 days (white arrowheads). GFP in green, blue indicates DAPI staining for DNA. Scale bars: 20 µm.

Biology Open • Supplementary information

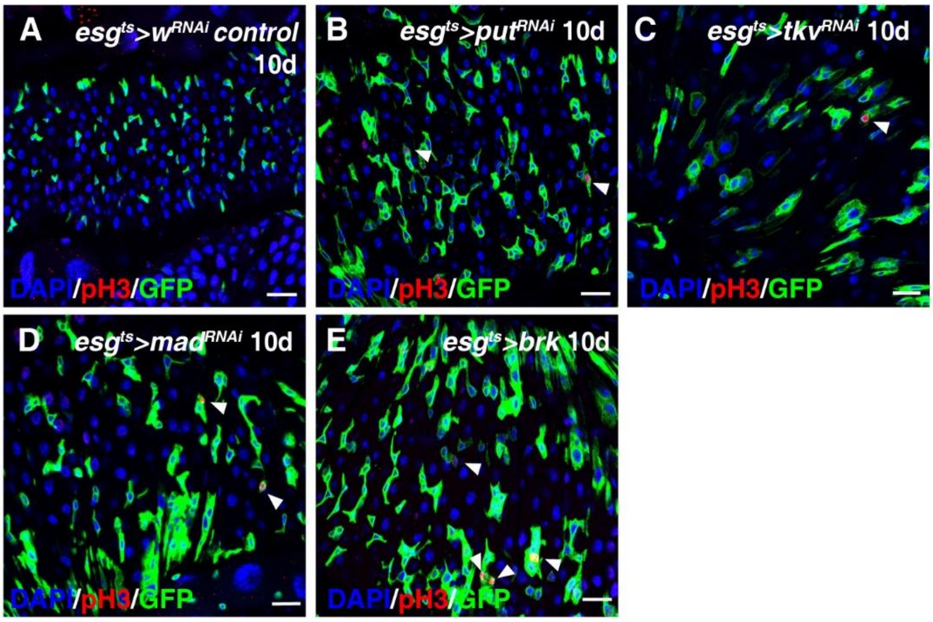

Fig. S8. ISC division is increased in the absence of Dpp signaling. (A) pH3 (red) in control intestines. (B) pH3 (red) in *esg*^{ts}>*put*^{RNAi} intestines (white arrowheads). (C) pH3 (red) in *esg*^{ts}>*tkv*^{RNAi} intestines (white arrowhead). (D) pH3 (red) in *esg*^{ts}>*mad*^{RNAi} intestines (white arrowheads). (E) pH3 (red) in *esg*^{ts}>*brk* intestines (white arrowheads). GFP in green, blue indicates DAPI staining for DNA. Scale bars: 20 µm. Please refer to Fig. 4G for quantification data.

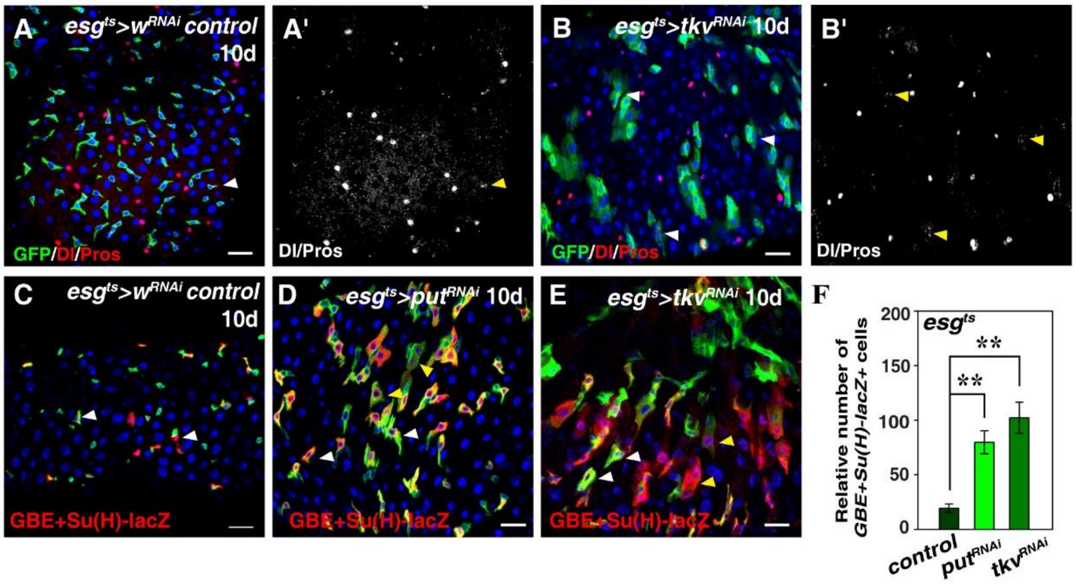

Fig. S9. Dpp signaling negatively regulates ISC proliferation and differentiation. (A) Dl and Pros (red) in control intestines at 29°C for 10 days (white arrowhead). Split channel for Dl and Pros (A', in black-white) in control intestines (yellow arrowhead). (B) Dl and Pros (red) in $esg^{ts}>tkv^{RNAi}$ intestines at 29°C for 10 days (white arrowheads). Split channel for Dl and Pros (B', in black-white) in $esg^{ts}>tkv^{RNAi}$ intestines (yellow arrowheads). (C) EBs (by *GBE+Su(H)-lacZ* in red) in control intestines at 29°C for 10 days (white arrowheads). (D) EBs (red) in $esg^{ts}>put^{RNAi}$ intestines at 29°C for 10 days (white arrowheads). Note that the size of the larger *GBE+Su(H)-lacZ*$^{+}$ cells is smaller compared to the size of the neighboring wildtype EC cells (polyploid *GBE+Su(H)-lacZ*$^{-}$ cells), indicating that Dpp signaling also affects EC maturation (yellow arrowheads). (E) EBs (red) in $esg^{ts}>tkv^{RNAi}$ intestines at 29°C for 10 days (white arrowheads). Note that the size of the larger *GBE+Su(H)-lacZ*$^{+}$ cells is smaller compared to the size of the neighboring wildtype EC cells (polyploid *GBE+Su(H)-lacZ*$^{-}$ cells), indicating that Dpp

Biology Open • Supplementary information

signaling also affects EC maturation (yellow arrowheads). (F) Quantification of the relative number of *GBE+Su(H)-lacZ*$^{+}$ cells in control, *esg*^{ts}*>put*^{RNAi}, and *esg*^{ts}*>tkv*^{RNAi} intestines. *n* = 10-15 intestines. mean ± SD is shown. $^{**}p < 0.01$. GFP in green, blue indicates DAPI staining for DNA. Scale bars: 20 µm.

Biology Open • Supplementary information

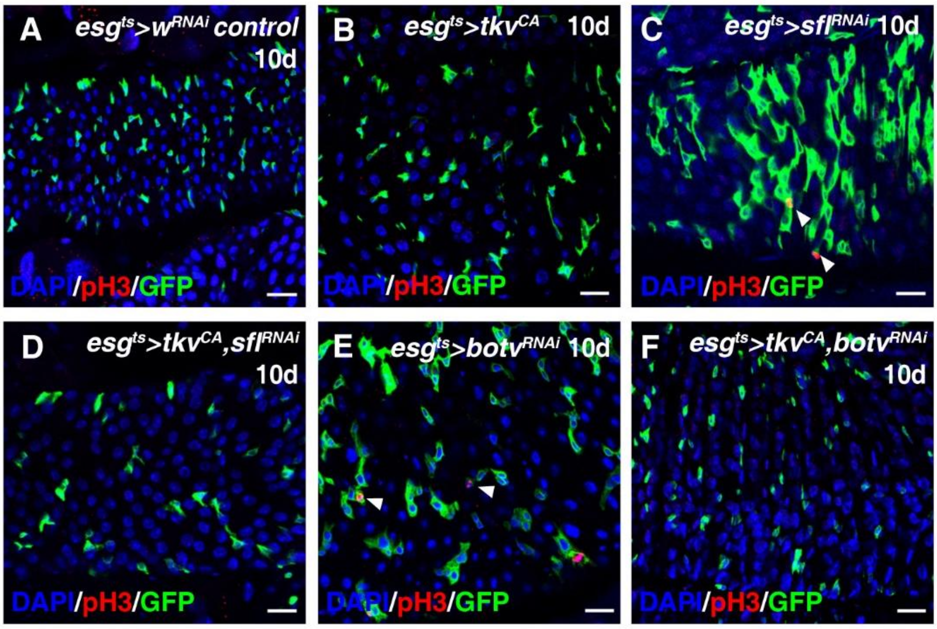

Fig. S10. Ectopic activation of Dpp signaling in progenitors completely rescued increased ISC proliferation in the absence of HS. (A) pH3 (red) in control intestines. (B) pH3 (red) in *esg*^{ts}>*tkv*^{CA} intestines. (C) pH3 (red) in *esg*^{ts}>*sfl*^{RNAi} intestines (white arrowheads). (D) pH3 (red) in *esg*^{ts}>*tkv*^{CA},*sfl*^{RNAi} intestines. (E) pH3 (red) in *esg*^{ts}>*botv*^{RNAi} intestines (white arrowheads). (F) pH3 (red) in *esg*^{ts}>*tkv*^{CA},*botv*^{RNAi} intestines. GFP in green, blue indicates DAPI staining for DNA. Scale bars: 20 µm. Please refer to Fig. 5H for quantification data.

Biology Open • Supplementary information

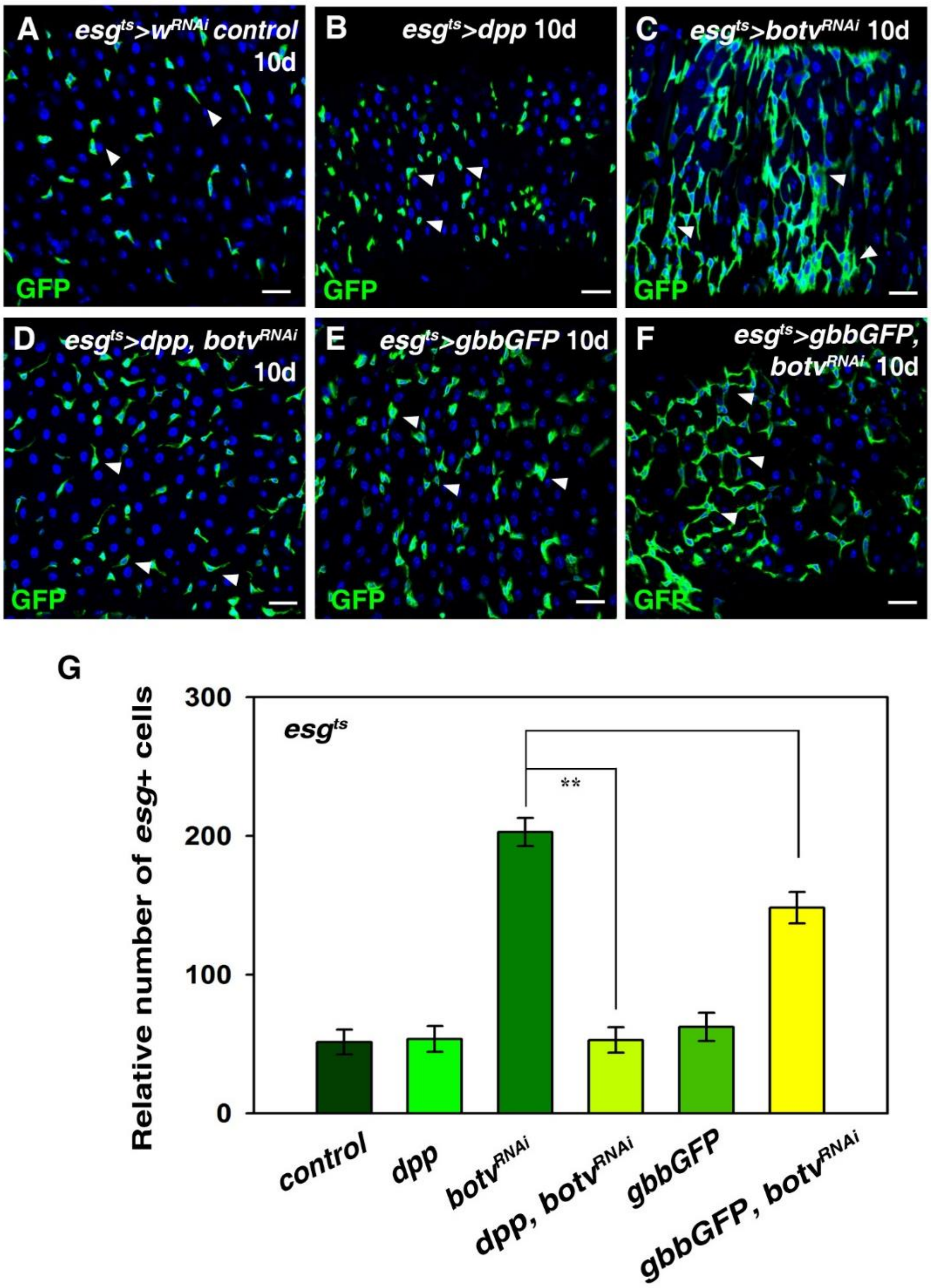

Fig. S11. Preference between Dpp and Gbb by HS.

(A) *esg*$^{+}$ cells (green) in control flies at 29°C for 10 days (white arrowheads). (B) *esg*$^{+}$ cells (green) in *esg*^{ts}>*dpp* flies at 29°C for 10 days (white arrowheads). (C) The number of *esg*$^{+}$ cells (green) is dramatically increased in *esg*^{ts}>*botv*^{RNAi} flies at 29°C for 10 days (white arrowheads). (D) The accumulation of *esg*+ cells in *esg*^{ts}>*botv*^{RNAi} flies is completely suppressed by co-expression of *UAS-dpp* at 29°C for 10 days (white

Biology Open • Supplementary information

arrowheads). (E) *esg*$^{+}$ cells (green) in *esg*^{ts}*>gbbGFP* flies at 29°C for 10 days (white arrowheads). (F) The accumulation of *esg*+ cells in *esg*^{ts}*>botv*^{RNAi} flies is only partially suppressed by co-expression of *UAS-gbbGFP* at 29°C for 10 days (white arrowheads). (I) Quantification of the relative number of *esg*$^{+}$ cells in different genotypes indicated. mean ± SD is shown. n = 10-15 intestines. $^{**}p< 0.01$. GFP in green, blue indicates DAPI staining for DNA. Scale bars: 20 μm.

Biology Open • Supplementary information

Table S1. qRT-PCR primers used

RpL11-F: GGTCCGTTCGTTCGGTATTCGC

RpL11-R: GGATCGTACTTGATGCCCAGATCG

sfl-F: GGGATCGCCACGTTTCATG

sfl-R: CCGCAGATCCTTCAGTGCCC

sgl-F: GCGCTGGATATCTACGATCCG

sgl-R: GTAGGCTGGCTTCATCATCG

ttv-F: GGATGCCGTTCTGTCGCTGG

ttv-R: GTGGTAGAATGCCGCCCCAG

sotv-F: GACAACTATGTGCTACCCTTCG

sotv-R: AGTACTTGGAGAACAACCAC

botv-F: GCGTATGCAGGCGAAGGAAG

botv-R: GGTGAACTGCTCCCGGGGG

Upd1-F: GGTGATGGACCGCTGATCCCAG

Upd1-R: CCGCAGCCTAAACAGTAGCCAGG

Upd2-F: CAAGTCTTTAGCTTCACCGCACTTGTG

Upd2-R: CAAGGACGAGTTATCAAGCGCAAGC

Upd3-F: ATCACCACCAATGCGGACAAGC

Upd3-R: TGGCCAGGTCCCAGTGCAACT
